# An introduction to the analysis of shotgun metagenomic data

**DOI:** 10.3389/fpls.2014.00209

**Published:** 2014-06-16

**Authors:** Thomas J. Sharpton

**Affiliations:** Department of Microbiology and Department of Statistics, Oregon State UniversityCorvallis, OR, USA

**Keywords:** metagenome, bioinformatics, microbiota, microbiome, microbial diversity, host–microbe interactions, review

## Abstract

Environmental DNA sequencing has revealed the expansive biodiversity of microorganisms and clarified the relationship between host-associated microbial communities and host phenotype. Shotgun metagenomic DNA sequencing is a relatively new and powerful environmental sequencing approach that provides insight into community biodiversity and function. But, the analysis of metagenomic sequences is complicated due to the complex structure of the data. Fortunately, new tools and data resources have been developed to circumvent these complexities and allow researchers to determine which microbes are present in the community and what they might be doing. This review describes the analytical strategies and specific tools that can be applied to metagenomic data and the considerations and caveats associated with their use. Specifically, it documents how metagenomes can be analyzed to quantify community structure and diversity, assemble novel genomes, identify new taxa and genes, and determine which metabolic pathways are encoded in the community. It also discusses several methods that can be used compare metagenomes to identify taxa and functions that differentiate communities.

## INTRODUCTION

Microorganisms are essentially everywhere in nature. Diverse communities of microbes thrive in environments ranging from the human gut (Walter and Ley, 2011), to the rhizosphere (Philippot et al., 2013), to conventionally inhospitable habitats such as acid mine runoff (Simmons et al., 2008) and geothermal hot springs (Sharp et al., 2014). Studies of cultured microbes reveal that they are critical components of these environments and provide essential ecosystem services (Arrigo, 2005; van der Heijden et al., 2008). Microbes that associate with a macroscopic host organism are no exception, and, in the subsequent discussion, are referred to as microbiota (note other definitions exist, e.g., Aminov, 2011). Microbiota can interact with their host to influence physiology and contribute to health, growth, or fitness (van der Heijden et al., 2008; Dimkpa et al., 2009; Hooper et al., 2012). For example, studies of model rhizosphere microbiota have taught us that they can impact plant growth (Kennedy et al., 2007), stress response (Redman et al., 2002; Yang et al., 2009), and pathogenic defense (Cook et al., 1995; Raupach and Kloepper, 1998). A comprehensive understanding of a macroscopic organism’s physiology requires investigation of its microbiota. Unfortunately, most microbes are notoriously difficult to culture in the laboratory.

Advances in DNA sequencing and biocomputing enable exploration of the genetic diversity of the uncultured component of host-associated microbial communities. Amplicon sequencing, for example, is the most widely used method for characterizing the diversity of microbiota. Here, a community is sampled (e.g., water, soil, tissue biopsy) and DNA is extracted from all cells in the sample. A taxonomically informative genomic marker that is common to virtually all organisms of interest is then targeted and amplified by PCR. The resultant amplicons are sequenced and bioinformatically characterized to determine which microbes are present in the sample and at what relative abundance. In the case of bacteria and archaea, amplicon sequencing studies usually target the small-subunit ribosomal RNA (16S) locus, which is both a taxonomically and phylogenetically informative marker (Pace et al., 1986; Hugenholtz and Pace, 1996). Amplicon sequencing of the 16S locus revealed an a tremendous amount of microbial diversity on Earth (Pace, 1997; Rappé and Giovannoni, 2003; Lozupone and Knight, 2007) and has been used to characterize the biodiversity of microbes from a great range of environments including the human gut (Human Microbiome Project Consortium, 2012a; Yatsunenko et al., 2012), *Arabidopsis thaliana* roots (Lundberg et al., 2012), ocean thermal vents (McCliment et al., 2006), hot springs (Bowen De León et al., 2013), and Antarctic volcano mineral soils (Soo et al., 2009). Comparing 16S sequence profiles across samples clarifies how microbial diversity associates with and scales across environmental conditions. In the case of microbiota, such observations have generated insight into host–microbe interactions and yielded hypotheses about microbiota-based disease mechanisms (Turnbaugh et al., 2009; Muegge et al., 2011; Bulgarelli et al., 2012; Smith et al., 2013). Follow-up microbiota-manipulation studies often confirm these hypotheses (Smith et al., 2013; David et al., 2014). Experimental design plays an important role in these analyses, as the most promising hypotheses tend to derive from comparisons of microbiota associated with cohorts of hosts of distinct genotypes or treatment conditions. (Kuczynski et al., 2011) provide a thorough review of how 16S amplicon sequencing can be used to study microbiota.

While powerful, amplicon sequencing is not without limitation. First, it may fail to resolve a substantial fraction of the diversity in a community given various biases associated with PCR (Hong et al., 2009; Sharpton et al., 2011; Logares et al., 2013). Second, amplicon sequencing can produce widely varying estimates of diversity (Jumpstart Consortium Human Microbiome Project Data Generation Working Group, 2012). For example, different genomic loci have differential power at resolving taxa (Liu et al., 2008; Schloss, 2010; Jumpstart Consortium Human Microbiome Project Data Generation Working Group, 2012). In addition, sequencing error and incorrectly assembled amplicons (i.e., chimeras), can produce artificial sequences that are often difficult to identify (Wylie et al., 2012). Third, amplicon sequencing typically only provides insight into the taxonomic composition of the microbial community. It is impossible to directly resolve the biological functions associated with these taxa using this approach. In some cases, phylogenetic reconstruction can be used to infer those biological functions that are encoded in a genome containing a particular 16S sequence (Langille et al., 2013). But, the accuracy with which these methods estimate the true functional diversity of a community is tied to how well the genomic diversity of the community is represented by the genomes available in sequence databases. Finally, amplicon sequencing is limited to the analysis of taxa for which taxonomically informative genetic markers are known and can be amplified. Novel or highly diverged microbes, especially viruses, are difficult to study using this approach. Additionally, because the 16S locus can be transferred between distantly related taxa (i.e., horizontal gene transfer), analysis of 16S sequences can result in overestimations of the community diversity (Acinas et al., 2004).

Shotgun metagenomic sequencing is an alternative approach to the study of uncultured microbiota that avoids these limitations. Here, DNA is again extracted from all cells in a community. But, instead of targeting a specific genomic locus for amplification, all DNA is subsequently sheared into tiny fragments that are independently sequenced. This results in DNA sequences (i.e., reads) that align to various genomic locations for the myriad genomes present in the sample, including non-microbes. Some of these reads will be sampled from taxonomically informative genomic loci (e.g., 16S), and others will be sampled from coding sequences that provide insight into the biological functions encoded in the genome. As a result, metagenomic data provides the opportunity to simultaneously explore two aspects of a microbial community: *who is there* and *what are they capable of doing?*

Despite these benefits, metagenomic sequence data presents several challenges. First, metagenomic data is relatively complex and large, complicating its informatic analysis. For example, it can be difficult to determine the genome from which a read was derived. Additionally, most communities are so diverse that most genomes are not completely represented by reads. As a result, two reads from the same gene may not overlap and are thus impossible to directly compare through sequence alignment (Schloss and Handelsman, 2008; Sharpton et al., 2011). When reads do overlap, it is not always evident if they are from the same or distinct genomes, which can challenge sequence assembly (Mavromatis et al., 2007; Mende et al., 2012). Also, metagenomic analysis tends to require a large volume of data to identify meaningful results because of the vast amount of genomic information being sampled. This requirement can pose computational problems. Fortunately, informatic software development is rapidly advancing and improving the ease and efficiency of metagenomic analysis. Second, metagenomes may contain unwanted host DNA, especially in the case of microbiota. In some situations, host DNA can so overwhelm community DNA that intricate molecular methods must be applied to selectively enrich microbial DNA prior to sequencing. Molecular and bioinformatic methods to filter host DNA from metagenomes either prior or subsequent to sequencing of the data are in development (Woyke et al., 2006; Chew and Holmes, 2009; Delmotte et al., 2009; Schmieder and Edwards, 2011b; Garcia-Garcerà et al., 2013). Third, while contamination is a challenge general to environmental sequencing studies (Degnan and Ochman, 2012), the identification and removal of metagenomic sequence contaminants is especially problematic (Kunin et al., 2008). For example, it can be difficult to determine which reads were generated from a detected contaminant’s genome. A metagenomic contaminant can mislead analyses of community genetic diversity if the contaminant’s genome is enriched for genes that are uncommon in the community, especially when the contaminant is highly abundant or has a large genome. Fortunately, software tools that identify and filter contaminant sequences in a metagenome exist (Schmieder and Edwards, 2011a). Finally, metagenomes tend to be relatively expensive to generate compared to amplicon sequences, especially in complex communities or when host DNA greatly outnumbers microbial DNA. Ongoing advances in DNA sequencing technology are improving the affordability of metagenomic sequencing.

These challenges have limited the application of metagenomic investigation. But, thanks to the aforementioned research advances, this analytical strategy has become more tractable for most laboratories. In recent years, metagenomic sequencing has been used to identify new viruses (Yozwiak et al., 2012), characterize the genomic diversity and function of uncultured bacteria (Wrighton et al., 2012), reveal novel and ecologically important proteins (Godzik, 2011), and identify taxa and metabolic pathways that differentiate gut microbiota associated with healthy and diseased humans (Morgan et al., 2012). The analysis of metagenomes has also been used to characterize plant microbiota, especially those associated with roots and leaves [as reviewed in Bulgarelli et al. (2013), Vorholt (2012)]. For example, metagenomic analysis has been used to identify physiological traits that differentiate rice leaf- and root-associating communities (Knief et al., 2012), characterize root endophytes of rice (Sessitsch et al., 2012), and quantify the physiological differences between microbiota associating with clover, soybean, and *Arabidopsis* leaves (Delmotte et al., 2009). The study of plant metagenomes can be difficult given that plants can have large genomes, which can overwhelm the genomic representation of the microbial community in the metagenome. Advances in laboratory procedures that physically separate microbiota from plant tissue (e.g., Jiao et al., 2006; Delmotte et al., 2009) will continue to improve the efficacy of metagenomic investigations in plant systems.

**FIGURE 1:**
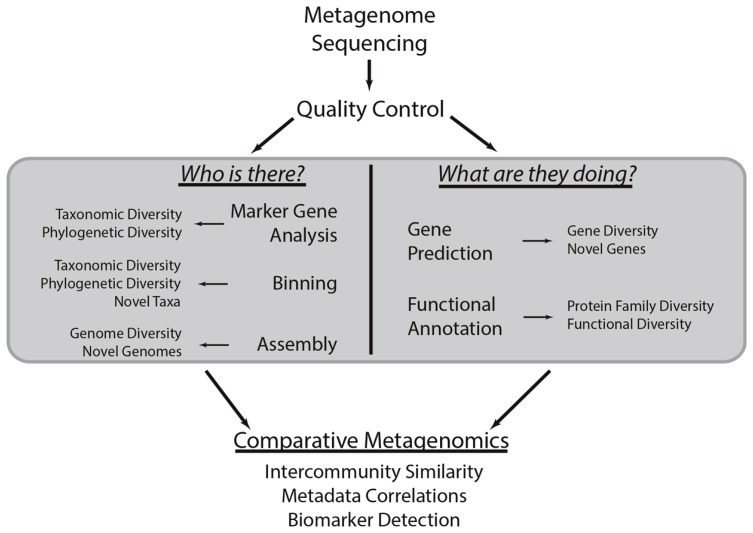
**Common metagenomic analytical strategies**. This methodological workflow illustrates a typical metagenomic analysis. First, shotgun metagenomic data is generated from a microbial community of interest. After conducting quality control procedures, metagenomic sequences can be subject to various analyses centered on the taxonomic and functional characterization of the community (gray box). These procedures are the focus of this review. Briefly, marker gene, binning, and assembly analyses provide insight into the taxonomic or phylogenetic diversity of the community and can identify novel taxa or genomes. Metagenomes can also be subject to gene prediction and functional annotation, which can be used to characterize the biological functions associated with the community and identify novel genes. The results of these various analyses can be compared to those obtained through analysis of other metagenomes to quantify the similarity between communities, determine how community diversity scales with environmental covariates (i.e., community metadata), and identify taxa and functions that stratify communities of various types (i.e., biomarker detection).

What follows in this review is a discussion of how metagenomic sequencing can be used to explore the taxonomic and functional diversity of microbial communities (**Figure 1**). It will briefly introduce analytical tools to this end, though it will not provide an exhaustive listing of all such tools. This review will assume that the reader is able to generate shotgun metagenomes, quality control the sequence data, and bioinformatically filter host DNA when relevant. The Human Microbiome Project standard operating protocols (http://www.hmpdacc.org/tools_protocols/tools_protocols.php) provide a thorough guide on how to conduct these steps (Human Microbiome Project Consortium, 2012a). Additional information on metagenomic analysis can be found in other reviews (e.g., Kunin et al., 2008; Kuczynski et al., 2011; Thomas et al., 2012; Davenport and Tümmler, 2013).

## *WHO IS THERE?* ASSESSING TAXONOMIC DIVERSITY

**FIGURE 2:**
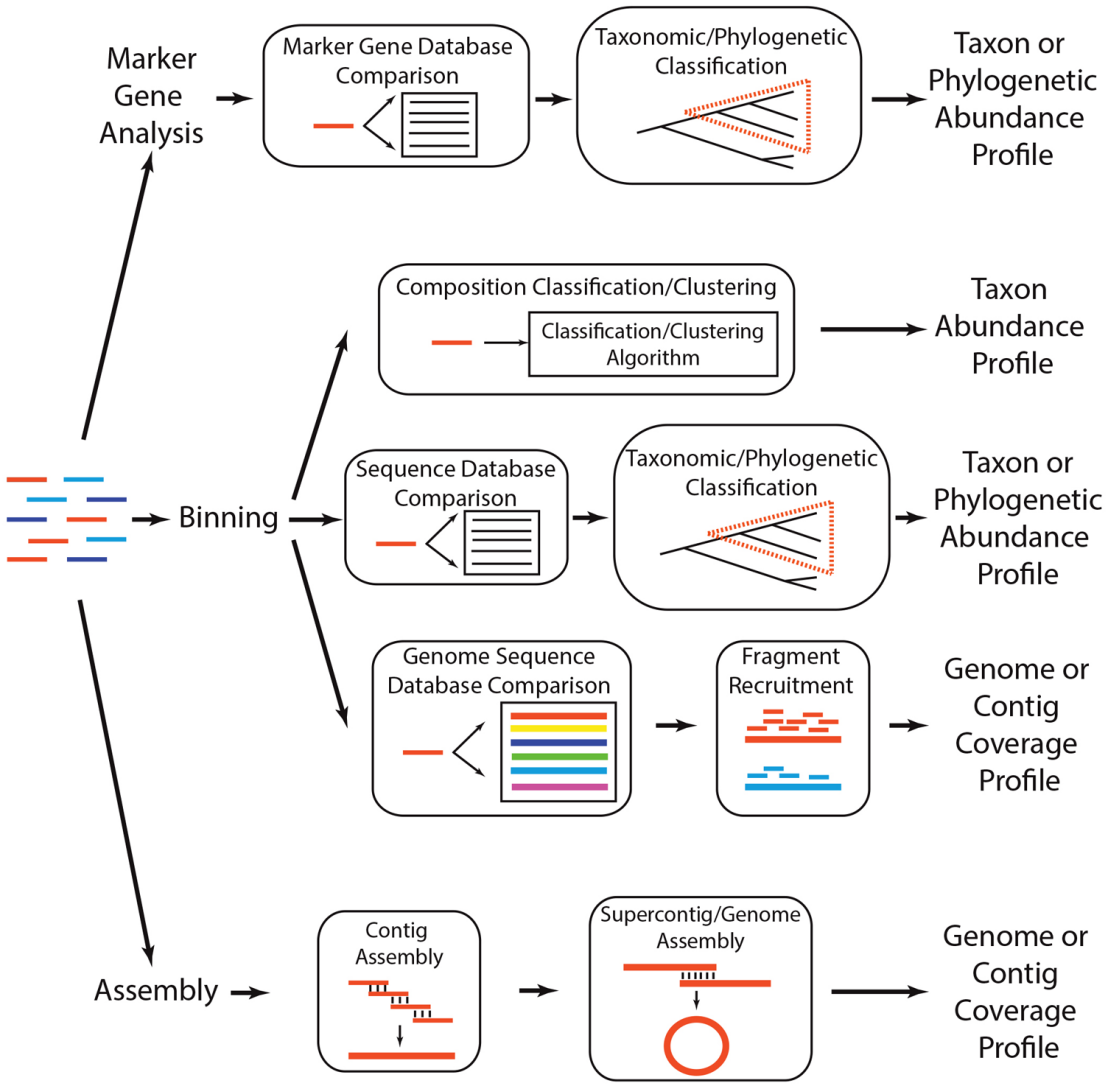
**Analytical strategies to determine which taxa are present in a metagenome**. A metagenome (colored lines, left) can be subject to three general analytical strategies that ultimately produce a profile of the taxa, phylogenetic lineages, or genomes present in the community. *Marker gene analyses* involve comparing each read to a reference database of taxonomically or phylogenetically informative sequences (i.e., marker genes), using a classification algorithm to determine if the read is a homolog of a marker gene, and annotating classified reads based on their similarity across marker gene sequences. There are several methods for *binning* metagenomes, including (1) compositional binning, which uses sequence composition to classify or cluster metagenomic reads into taxonomic groups, (2) similarity binning, which classifies a read into a taxonomic or phylogenetic group based on its similarity to previously identified genes or proteins, and (3) fragment recruitment, wherein reads are aligned to nearly identical genome sequences to produce metagenomic coverage estimates of the genome. Finally, sequences can be subject to *assembly*, wherein reads that share nearly identical sequence at their ends are merged to create contigs, which can subsequently be assembled into supercontigs or complete genomes.

One of the primary ways by which a microbial community can be characterized is the quantification of its taxonomic diversity. This involves determining which microbes are present in a community (i.e., richness) and at what abundance. Taxonomic diversity serves as a way of profiling a community and can be used to ascertain the similarity of two or more communities (e.g., communities with more shared taxa are more similar). Additionally, taxonomic diversity may provide some insight into the biological function of the community when it contains members of functionally described taxa (e.g., the presence of Cyanobacteria suggests that the community is photosynthetic). In the case of metagenomics, taxonomic diversity is typically quantified by either (1) analyzing taxonomically informative marker genes, (2) grouping sequences into defined taxonomic groups (i.e., binning), or (3) assembling sequences into distinct genomes. These approaches are not mutually exclusive and may be synergistic (**Figure 2**). For example, in some situations, it may be appropriate to bin sequences into taxonomic groups and then subject each group’s sequences to assembly, while other cases may warrant conducting an initial assembly and then subjecting the assembled sequences to binning.

### MARKER GENE ANALYSIS

Marker gene analysis is one of the most straightforward and computationally efficient ways of quantifying a metagenome’s taxonomic diversity. This procedure involves comparing metagenomic reads to a database of taxonomically informative gene families (i.e., marker genes), identifying those reads that are marker gene homologs, and using sequence or phylogenetic similarity to the marker gene database sequences to taxonomically annotate each metagenomic homolog. The most frequently used marker genes include rRNA genes or protein coding genes that tend to be single copy and common to microbial genomes. Because this approach involves comparing metagenomic reads to a relatively small database for the purpose of a similarity search (e.g., not all gene families are taxonomically informative), marker gene analysis can be a relatively rapid way to estimate the diversity of a metagenome. Additionally, focusing on single-copy gene families may provide more accurate estimates of taxonomic abundance than methods that consider families known to widely vary in copy number across genomes (e.g., similarity based binning, below; Liu et al., 2011). This general strategy may be applied to assembled or unassembled reads, though some specific methods may only be applicable to one of these two data types.

There are two general methods by which marker genes are used to taxonomically annotate metagenomes. The first relies on sequence similarity between the read and the marker genes. For example, MetaPhyler uses the results of a pairwise sequence search between metagenomic reads and a database of marker genes as well as a series of custom classifiers that are considerate of family (e.g., rate of evolution) and read (e.g., sequence length) properties to determine the taxonomy of the metagenomic sequence (Liu et al., 2011). MetaPhlAn also relies on sequence similarity to taxonomically characterize metagenomic marker gene homologs. It uses an extensive database of phylogenetic clade-specific markers (i.e., families that are single copy and generally only common to a monophyletic group of taxa) to assign metagenomic sequences to specific taxonomic groups (Segata et al., 2012). The second approach uses phylogenetic information, which may take longer to calculate, but may also provide greater accuracy. For example, AMPHORA (Wu and Eisen, 2008; Wu and Scott, 2012), uses hidden Markov models (HMMs) to identify metagenomic homologs of phylogenetically informative, single copy protein-coding genes that are common to sequenced genomes from either bacteria or archaea. It then assembles a marker gene phylogeny that includes metagenomic homologs, which are annotated based on their relative location in the tree (i.e., phylotyping). PhyloSift (Darling et al., 2014) is similar, but uses an expanded marker database, including an extensive viral gene family database, and edge PCA (Matsen and Evans, 2013) to identify specific lineages in a marker gene’s phylogenetic tree that differ between communities. PhylOTU uses a phylogenetic tree to relate non-overlapping metagenomic 16S homologs, which are subsequently clustered into taxonomic groups based on phylogenetic distance (Sharpton et al., 2011).

There are several important caveats to be aware of when using marker genes to analyze metagenomes. First, this strategy operates under the assumption that the relatively small fraction of the metagenome that is homologous to marker genes represents an accurate sampling of the entire taxonomic distribution of the community. While researchers take great effort to identify marker genes that are uniformly present across clades of genomes, the genome sequences available to researchers during marker gene identification may not adequately reflect the diversity of genomes present in the community under investigation. Second, marker gene analysis is not appropriate for taxa that do not contain the markers being explored. Thanks to recent efforts to identify phylogenetic clade-specific marker genes (Segata et al., 2012; Wu et al., 2013; Darling et al., 2014) and expand the phylogenetic diversity represented in genome sequence databases (Wu et al., 2009), this may be a diminishing problem. Third, the accuracy of annotation is based on properties of the marker family and likely varies across markers. Accuracy is also a function of how well the reference database reflects the community under investigation. Expanding the phylogenetic diversity of available genomes sequences can mitigate these limitations.

### BINNING

A related strategy, known as binning, attempts to assign every metagenomic sequence to a taxonomic group. Generally, each sequence is either (1) classified into a taxonomic group (e.g., OTU, genus, family) through comparison to some referential data or (2) clustered into groups of sequences that represent taxonomic groups based on shared characteristics (e.g., GC content). Binning plays an important role in the analysis of metagenomes. First, depending on the method used, binning may provide insight into the presence of novel genomes that are difficult to otherwise identify. Second, it provides insight into the distinct numbers and types of taxa in the community. While many approaches provide a coarse resolution of taxonomy, some are capable of indicating strain-level variation, though usually at the expense of fewer binned reads. Third, binning provides a way of reducing the complexity of the data, such that post-binning analyses (e.g., assembly) can be performed independently on each set of binned reads rather than on the entire population of data. Binning may be conducted on assembled or unassembled data, though most algorithms report that binning accuracy improves as sequence lengths increase. Binning algorithms generally come in one of three flavors: sequence composition, sequence similarity, and fragment recruitment.

Sequence compositional binning uses metagenome sequence characteristics (e.g., tetramer frequency) to cluster or classify sequences into taxonomic groups. These methods generally do not require the alignment of reads to a reference sequence database and, as a result, can process large metagenomes relatively rapidly. Some of these methods instead analyze whole genome sequences ahead of time to train classifiers that stratify sequences into taxonomic groups. For example, PhyloPithia and PhylopithiaS (McHardy et al., 2007; Patil et al., 2011) use support vector machines, which analyze training sequences associated with various phylogenetic groups to build oligonucleotide frequency models that determine whether a new sequence (e.g., a metagenomic read) is a member of the group. A related tool, Phymm (Brady and Salzberg, 2009; Brady and Salzberg, 2011), uses interpolated Markov models (Salzberg et al., 1998), which combine prediction probabilities derived from a variety of training sequence oligonucleotide lengths, and, optionally, blast search results, to classify metagenomics reads into phylogenetic lineages. Other methods use sequence characteristics to cluster metagenomic reads into distinct groups without querying a reference database, and thus may identify previously unknown organisms. For example, emergent self-organizing maps (ESOMs) can be used to cluster assembled metagenomic reads based on tetranucleotide frequency and, optionally, contig coverage and abundance distributions (Dick et al., 2009). While taxonomic annotations are not identified directly from this approach, it has proven useful for partitioning contigs into groups that can be subsequently assembled into nearly complete genomes representing uncharacterized organisms (Wrighton et al., 2012). Two-tiered clustering (Saeed et al., 2012) is a related approach that first bins sequences into coarse groups based on GC content and the oligonucleotide frequency-derived error gradient (Saeed and Halgamuge, 2009), which assesses the variance in oligonucleotide frequency across the length of a read, and then subdivides these initial clusters based on tetranucleotide frequency. There are many additional compositional binning algorithms – including NBC, a naïve bayes classifier that has been shown to annotate more sequences than some sequence-similarity based procedures (Rosen et al., 2008) – and listing them all is beyond the scope of this review. While compositional binning algorithms have proven useful for the analysis of metagenomes, they generally operate under the assumption that the sequence characteristics being interrogated tend to be phylogenetically informative. Variation in the taxonomic bias of these sequence characteristics may result in inaccurate assignments for a fraction of the data. Also, the accuracy of these methods, especially the classifiers, is tied to the selection of genomes used to train the classification algorithm.

Metagenomic reads can also be binned based on their sequence similarity to a database of taxonomically annotated sequences. Compared to compositional binning tools, these methods tend to require greater computational resources as every read is usually aligned to a large volume of sequences. In addition, these methods, like classification-based compositional binning algorithms, are not necessarily ideal for the identification of novel genomes, though they may be used to identify phylogenetic nodes that contain putatively novel lineages. That said, similarity based methods may provide higher annotation accuracy and resolution compared to compositional binning. One of the most widely used tools is MEGAN, which is a sequence similarity approach that uses blast to compare metagenomic reads to a database of sequences that are annotated with NCBI taxonomy (Huson et al., 2011). It then infers the taxonomy of the sequence by placing the read on the node in the NCBI taxonomy tree that corresponds to the last common ancestor of all the taxa that contain a homolog of the read. MG-RAST is also widely used; it uses phylogenomic reconstruction of database sequences to which a read is similar to infer the read’s taxonomy (Meyer et al., 2008). CARMA uses reciprocal best hits between database sequences and metagenomic reads and models gene-family specific rates of evolution to infer the appropriate taxonomic rank of each metagenomic read (Gerlach and Stoye, 2011). Note that while MetaPhlAn and PhyloSift were introduced in the marker gene analysis section, one may consider these types of methods as binning algorithms, especially as the database of marker genes expands.

A related approach, called fragment recruitment, identifies reads that exhibit nearly identical alignments to genome sequences (i.e., read mapping) and partitions reads based on the genome to which they map. This approach was used to taxonomically characterize reads in the global ocean survey Rusch et al. (2007) and Qin et al. (2010) used it to estimate the abundance of gut microbiota in healthy and inflammatory bowel diseased individuals. MOSAIK (Lee et al., 2013) was used by Schloissnig et al. (2013) to map reads to microbial genomes for the purpose of characterizing strain-level variation in the human microbiome. Fragment recruitment can also be used at the level of genes to quantify the abundance of metabolic pathways (Desai et al., 2013; see Gene Prediction). There are currently few tools that will handle both the mapping of reads to a database of genomes and the calculation of genome abundance. Genometa (Davenport et al., 2012) is one such tool and provides a graphical user interface. (Martin et al., 2012) evaluated the performance of several commonly used short read mapping algorithms (e.g., SOAP, BWA, CLC) in fragment recruitment using RefCov, which analyzes the output files produced by these algorithms and calculates recruitment statistics such as coverage depth and breadth. These methods are not necessarily ideal for communities that contain genomes outside of the scope of genome sequences in reference databases and are not useful for the analysis of novel taxa.

There are several general caveats associated with binning. First, there is usually a trade-off between the number of reads that are binned and the taxonomic specificity of the annotations assigned to each bin. Additionally, while binning provides a way of annotating a substantial fraction of the metagenome, there may be large variance in the accuracy and specificity of the annotations across reads. Second, convergent evolutionary characteristics, including horizontal gene transfer, may diminish the accuracy of binning, especially for composition-based approaches and for the study of those taxa that may not be well-represented by the training data. Finally, in the case of novel organisms, it is often difficult to validate an algorithm’s predictions. Multiple independent predictions of the organism’s existence (e.g., different algorithms, different communities) can provide additional support, but subsequent experimental verification may be necessary.

### ASSEMBLY

Assembly merges collinear metagenomic reads from the same genome into a single contiguous sequence (i.e., contig) and is useful for generating longer sequences, which can simplify bioinformatic analysis relative to unassembled short metagenomic reads. In some instances, complete or nearly complete genomes can be assembled, which provides insight into the genomic composition of uncultured organisms found in a community (Iverson et al., 2012; Wrighton et al., 2012; Ruby et al., 2013). If used to quantify taxonomic abundance, one must be careful to track contig coverage (i.e., the number of assembled reads that align to the average base in the contig), as contigs are subsequently treated as a single sequence in most downstream analyses, and analytical tools may thus not accurately quantify the abundance of the taxon as it is represented in the raw data. The major challenge associated with assembly is the generation of chimeras, wherein sequences from two distinct genomes are spuriously assembled into a contig due to shared sequence similarity. Chimeras are more likely to be generated in relatively complex communities (Luo et al., 2012), so researchers often bin reads and independently assemble each bin to mitigate the risk of generating chimeras.

While there are many algorithms for assembling nucleic acid sequences, relatively few have been designed to deal with the specific informatic challenges associated with metagenomes. Many tools build upon the traditional de Brujin graph approach to genome assembly [thoroughly reviewed in Compeau et al. (2011)], wherein a network (i.e., graph) models the contiguous sequence overlap between all subsequences of a specified length (i.e., *k-mers*) in a read as well as the corresponding *k-mers* in all other reads that are linked through overlapping sequence identity. For example, tools like MetaVelvet (Namiki et al., 2012) and Meta-IBDA (Peng et al., 2011) generate a de Brujin graph from the entire metagenome and use properties of the graph or sequence data to identify sub-graphs that represent genome-specific assemblies. Genovo constructs a probabilistic model of assembly and outputs the set of contigs with the highest likelihood (Laserson et al., 2011). Because of the complexity associated with *de novo* metagenome assembly, several recent tools implement data reduction or efficiency procedures to reduce the amount of memory or time needed to complete assembly. These tools may be the only options for those labs without sophisticated computing environment (e.g., big memory machines). For example, diginorm (Brown et al., 2012) filters redundant reads by normalizing the distribution of *k-mers* in a metagenome. Another tool, khmer, stores the nodes of a de Brujin graph in a memory-efficient structure (McDonald and Brown, 2013). PRICE implements a series of data reduction procedures to minimize the complexity associated with generating an initial set of contigs and then uses paired-end information associated with reads to merge contigs (Ruby et al., 2013). Ray Meta (Boisvert et al., 2012) uses a distributed computing environment (e.g., a cloud or computer cluster) to disburse the computationally expensive task of assembly across multiple computers, which improves the rate at which massive sequence libraries can be assembled. MetAMOS is a modular workflow that executes a variety of assembly algorithms and conducts taxonomic and functional annotation on the resulting contigs (Treangen et al., 2013).

There are several considerations associated with assembling metagenomic sequences. First, assembly tends to be limited to the most abundant taxa in the community. Without extensive sequencing, it may be difficult to assemble genomes of rare microbiota. Second, assembly may produce *in silico* chimeras, so it should be used cautiously and with consideration. Repetitive regions within a genome are also notoriously difficult to assemble; analysis of repeat copy number variation from assemblies should be carefully evaluated. Combining long-read (e.g., Pacific Bioscience) and short-read (e.g., Illumina) sequences in the same assembly may limit these errors, though there are currently few tools that can combine these types of data (Deshpande et al., 2013). Third, assembly can be computationally intensive, especially in its requirements for RAM. In addition to the efficiency tools mentioned above, binning sequences prior to assembly can be a good way to cut down on the computational complexity.

## *WHAT ARE THEY CAPABLE OF DOING?* INFERRING BIOLOGICAL FUNCTION

**FIGURE 3:**
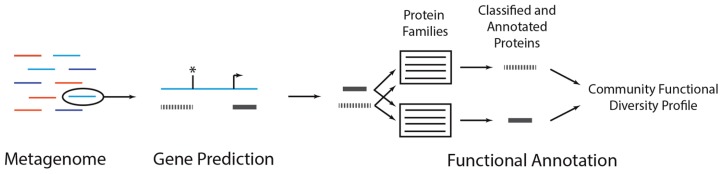
**A metagenomic functional annotation workflow**. A metagenome (colored lines, left) can be annotated by subjecting each reads to gene prediction and functional annotation. In *gene prediction*, various algorithms can be used to identify subsequences in a metagenomic read (blue line) that may encode proteins (gray bars). In some situations, coding sequences may start (arrow) or stop (asterisk) upstream or downstream the length of the read, resulting in partial gene predictions. Each predicted protein can then be subject to *functional annotation*, wherein it is compared to a database of protein families. Predicted peptides that are classified as homologs of the family are annotated with the family’s function. Conducting this analysis across all reads results in a community functional diversity profile. As discussed in the main text, there are alternative annotation strategies and variations on this general procedure.

Metagenomes provide insight into a community’s physiology by clarifying the collective functions that are encoded in the genomes of the organisms that make up the community. The functional diversity of a community can be quantified by annotating metagenomic sequences with functions (**Figure 3**). This usually involves identifying metagenomic reads that contain protein coding sequences and comparing the coding sequence to a database of genes, proteins, protein families, or metabolic pathways for which some functional information is known. The function of the coding sequence is inferred based on its similarity to sequences in the database. Doing this for all metagenomic sequences produces a profile that describes the number of distinct types of functions and their relative abundance in the metagenome. This profile can be used to compare metagenomes to identify those communities that are metabolically similar (Human Microbiome Project Consortium, 2012b), ascertain how various treatments influence the functional composition of the community (Looft et al., 2012), and reveal those functions that associate with specific environmental or host-physiological variables (i.e., biomarkers) and may be useful for environmental or host diagnosis (Morgan et al., 2012). Metagenomes may also reveal the presence of novel genes (Nacke et al., 2012) or provide insight into the ecological conditions associated with those genes for which the function is currently unknown (Buttigieg et al., 2013). In general, metagenome functional annotation involves two non-mutually exclusive steps: gene prediction and gene annotation.

### GENE PREDICTION

Gene prediction determines which metagenomic reads contain coding sequences. Once identified, coding sequences can be functionally annotated. Gene prediction can be conducted on assembled or unassembled metagenomic sequences. For assembled metagenomes with full-length coding sequences, gene prediction is akin to the framework used during the analysis of whole genome sequences, with the caveat that some prediction algorithms require species-specific parameters that may not always be appropriate when the contigs have been sampled from diverse or novel lineages. For example, many gene prediction algorithms are typically trained using sequence features of closely related organisms. An extensive review on gene prediction in assembled genomes is covered by Yandell and Ence (2012), Richardson and Watson (2013). For unassembled or poorly assembled metagenomes, the problem is more challenging and involves predicting partial coding sequences, in the case that a gene starts upstream or stops downstream of the length of the read. There tend to be three ways by which genes are predicted in metagenomes: (1) gene fragment recruitment, (2) protein family classification, and (3) *de novo* gene prediction. Note that because of the considerable diversity of genomes in nature compared to those in sequence databases (Wu et al., 2009), not all predicted genes will exhibit homology to known sequences. Some of these predictions may be spurious, while others will represent novel or highly diverged proteins. Thus, gene prediction is not just an important step in functional annotation, but is critical to the identification of novel genes.

One of the most straightforward ways of identifying coding sequences in a metagenome is to use fragment recruitment (see Binning) to map metagenomic reads or contigs to a database of gene sequences. Metagenomic sequences that are identical or nearly identical to a full-length gene sequence are considered representative subsequences of the gene. In the case that the gene has a functional annotation, this method of gene prediction can also simultaneously provide a functional annotation for the recruited metagenomic sequences (Desai et al., 2013). This procedure has been used to quantify the genetic diversity of marine communities (Rusch et al., 2007) and gut microbiota (Qin et al., 2010; Human Microbiome Project Consortium, 2012a), and is generally useful for cataloging the specific genes present in the metagenome. This is generally a high-throughput gene prediction procedure because it tends to rely on read mapping algorithms that rapidly assess whether a genomic fragment is nearly identical to a database sequence. However, this comes at the expense of being able to identify diverse homologs of a known gene. As a result, it may not be the most appropriate gene prediction procedure for metagenomes derived from communities with genomes that are underrepresented in sequence databases, especially if the identification of novel or highly divergent genes is desired.

A related approach involves translating each metagenomic read into all six possible protein coding frames and comparing each of the resulting peptides to a database of protein sequences by sequence alignment. The alignments can then be analyzed to identify those metagenomic sequences that encode translated peptides that exhibit homology to proteins in the database. This can be conducted by using sequence translation tools like transeq (Rice et al., 2000) to translate reads prior to conducting protein sequence alignment using blastp or fast blast algorithms like USEARCH (Edgar, 2010), RAPsearch (Zhao et al., 2012), or lastp (Kiełbasa et al., 2011). Alternatively, alignment algorithms that translate nucleic acid sequences on the fly, like blastx (Altschul et al., 1997), USEARCH with the ublast option, or lastx (Kiełbasa et al., 2011) can be used. This gene prediction procedure is most frequently used in concert with functional annotation, wherein the annotation of the protein sequence to which the translated metagenomic read is homologous is used to infer the read’s annotation (discussed in more depth below). Since this method relies on comparing metagenomic sequences to a reference database of known sequences, it is not useful for identifying novel types of proteins. But, it can reveal diverged homologs of known proteins.

*De novo* gene prediction, on the other hand, can potentially identify novel genes. Here, gene prediction models, which are trained by evaluating various properties of microbial genes (e.g., length, codon usage, GC bias), are used to assess whether a metagenomic read or contig contains a gene and does not rely on sequence similarity to a reference database to do so. As a result, these methods can identify genes in the metagenome that share common properties with other microbial genes but that may be highly diverged from any gene that has been discovered to date. There are several tools that can be used for *de novo* gene prediction, including MetaGene (Noguchi et al., 2006), Glimmer-MG (Kelley et al., 2012), MetaGeneMark (Zhu et al., 2010), FragGeneScan (Rho et al., 2010), Orphelia (Hoff et al., 2009), and MetaGun (Liu et al., 2013). In Trimble et al. (2012), many of these methods were compared using statistical simulations. The authors found that their performance varied as a function of read properties (e.g., length and sequencing error rate), with different methods producing optimal accuracies at different property thresholds, which suggests that researchers need to be careful about selecting the appropriate algorithm for their data. As in genome annotation, Yok and Rosen (2011) found that gene prediction in metagenomes improves when multiple methods are applied to the same data (e.g., a consensus approach). While these methods can require a fair bit of time and resources to predict genes, they tend to be more discriminating than 6-frame translation and may save time when functionally annotating sequences as fewer pairwise sequence comparisons may be necessary (Trimble et al., 2012). In the case that the predicted gene is novel relative to database sequences, it can be difficult to determine if the gene is real or a spurious prediction. Identifying homologs of the gene in other communities may be one way of reinforcing *de novo* predictions.

### FUNCTIONAL ANNOTATION BY PROTEIN FAMILY CLASSIFICATION

Once coding sequences in a metagenome are predicted, they can be subject to functional annotation. The most common way this is accomplished is by classifying the predicted metagenomic proteins into protein families. A protein family is a group of evolutionarily related protein sequences, or subsequences in the case of protein domain families (e.g., Pfam; Finn et al., 2014). They are usually characterized by comparing full-length protein sequences that have been identified through genome sequencing projects. Because the proteins in a family share a common ancestor, they are thought to encode similar biological functions. If a metagenomic sequence is determined to be a homolog of this family (i.e., it is classified as being a member of the family), then it is inferred that the sequence encodes the family’s function. Classification of an assembled or unassembled metagenomic protein sequence into a protein family usually requires comparing the metagenomic protein to either a database of protein sequences, each of which is designated as being a member of a family, or comparison of the sequence to a probabilistic model that describes the diversity of proteins in the family (e.g., HMMs). Once the metagenomic sequence has been compared to all proteins or all models, it can either be classified into (1) a single family (e.g., the family with the best hit), (2) a series of families (e.g., all families that exhibit a significant classification score), or (3) no family, which suggests that the protein may be novel, highly diverged, or spurious. There are exceptions to this annotation framework, such as the gene recruitment procedure mentioned in the Gene Prediction section, though they are less commonly used.

There are many databases that can be used to functionally annotate metagenomic proteins. They generally come in two varieties: sequence databases and HMM databases. Comparing metagenomic reads to a database of sequences tends to be relatively fast and may produce more specific hits for reads that are closely related to sequences in the database, whereas comparing metagenomic reads to a database of HMMs tends to identify more distantly related and diverged members of a family, though their precision for very short sequences is not well explored. Commonly used sequence databases include the SEED annotation system, which is employed by MG-RAST and links specific family level functions into higher-order functional subsystems (Overbeek et al., 2014). KEGG orthology groups have proven to be particularly useful as they conveniently map to KEGG metabolic pathway modules (Kanehisa et al., 2014). MetaCyc is similar in that the families are mapped to highly curated and well-described metabolic pathways, though their reliance on functional precision comes at the expense of database sequence diversity (Caspi et al., 2014). EggNOG is a database of non-supervised orthologs groups of proteins that tends to be frequently updated so as to include a relatively large amount of sequence diversity (Powell et al., 2014). The use of HMM databases in metagenomic analyses tends to be limited to Pfam, which uses HMMs to model protein domains (Finn et al., 2014). Recent years have seen the generation of databases of HMMs of full-length and phylogenetically diverse protein families. This includes Phylofacts (Afrasiabi et al., 2013) and the SiftingFamilies database (Sharpton et al., 2012), which, like EggNOG, tend to be frequently updated.

Protein family classification of metagenomic reads tends to require substantial computing resources because all metagenomic peptides are compared to all protein sequences or models in the database. Fortunately, each comparison is independent, so computing clusters and multi-core servers can distribute the computational load in parallel to improve throughput. There are several web servers that interface with distributed computing clusters to conduct gene prediction, the database search, family classification and annotation. These include MG-RAST (Meyer et al., 2008), CAMERA (Sun et al., 2011), and IMG/M (Markowitz et al., 2014). These tools tend to be relatively easy to use, though they do place some constraints on the analysis (e.g., the protein family database). As an added benefit, these resources provide public access to many metagenomes and comparative metagenomic tools. There are also standalone workflows that researchers can install on their own systems, such as RAAMCAP (Li, 2009), SmashCommunity (Arumugam et al., 2010) and MetAMOS (Treangen et al., 2013), which often provide more analytical flexibility. The Human Microbiome Project data was annotated using HUMAnN (Abubucker et al., 2012), which maps metagenomic reads to KEGG pathways to produce pathway coverage and abundance profiles. There are also post-processing tools that analyze protein family classification results produced independently by the researcher. For example, ShotgunFunctionalizeR (Kristiansson et al., 2009) is an R package that enables comparative metagenomic analyses including the identification of families and pathways that are overrepresented in particular samples or that correlate with particular sample properties (e.g., environmental conditions). Similarly, LefSe (Segata et al., 2012) conducts robust statistical tests to identify those taxa, genes, or pathways that stratify two or more metagenomes.

While protein family classification of metagenomic reads is a useful way of inferring community function, it is imperfect. First, the functional diversity encoded in the metagenome may only approximate the community’s functional activity. The presence of a gene does not mean that it is expressed at the time of sampling. That said, comparative metagenomic and metatranscriptomic analyses indicate that differences between communities at the transcriptional level are often mirrored at the genomic level, suggesting that metagenomes may provide a meaningful proxy for activity (Mason et al., 2012). Additionally, the detection of enriched functions in a metagenome suggests that they are important to some aspect of the dynamic interaction between the community and its environment or host. Analysis of metatranscriptomic and metaproteomic data can provide additional insight into which pathways are actively expressed in the community, though they provide lower coverage of the functions found in the community (Gilbert et al., 2010; Simon and Daniel, 2011; Mason et al., 2012). Second, most databases contain families that have no known functional annotation. Metagenomic reads that are determined to be homologs of such families will not be ascribed a function. These families can still be informative, as they can provide support for metagenomic coding sequence predictions and may be useful diagnostics. Third, the protein family database used to annotate the sequences may be subject to phylogenetic biases, such that certain communities are disproportionately more accurately or more thoroughly annotated than others (Wu et al., 2009). Each database also uses different approaches for identifying families and functionally annotating them. The result is that different databases may annotate different proportions of the metagenome and may produce different functional profiles that describe the community. Fourth, this method presumes that function is relatively evolutionarily static. Evolutionarily plastic functions erode the specificity with which function can be inferred. Finally, there may be more proteins and functions in nature than those that have been described by current sequence databases (Wu et al., 2009; Godzik, 2011). Novel strategies for functionally annotating metagenomes and improvements in the way predicted metagenomic proteins are integrated into protein family databases are needed.

## CONCLUSION

Researchers interested in analyzing metagenomes to characterize microbial community diversity and function now have a litany of tools and data resources at their disposal. Many of the tools discussed here were developed for researchers comfortable interfacing with a command-line environment. This is understandable given the complexity of metagenomic data and the computational requirements traditionally associated with its analysis. But, many researchers interested in metagenomic analysis may not have experience working with this type of software or access to the necessary computational resources. Fortunately, there are many web-based tools that centralize metagenome data management and analysis and provide researchers with the means to annotate and compare metagenomes through an easy-to-use interface (**Table 1**). These tools will not necessarily conduct all analytical strategies and frequently do not provide the flexibility and customization of their command-line counterparts.

**Table 1:** Web-based metagenomic analysis resources.

Resource	Methods	Citation	Web link
AmphoraNet	Marker gene analysis: phylogeny	Kerepesi et al. (2014)	http://pitgroup.org/amphoranet/
CAMERA	Various: taxonomic and functional annotation, comparative analyses	Sun et al. (2011)	http://camera.calit2.net/
Comet	Functional annotation, comparative analyses	Lingner et al. (2011)	http://comet.gobics.de/
LEfSe (Galaxy)	Comparative analyses	Segata et al. (2011)	http://huttenhower.sph.harvard.edu/galaxy/
IMG/M	Various: taxonomic and functional annotation, comparative analyses	Markowitz et al. (2014)	https://img.jgi.doe.gov/m/
MG-RAST	Various: taxonomic and functional annotation, comparative analyses	Meyer et al. (2008)	http://metagenomics.anl.gov/
MALINA	Various: taxonomic and functional annotation, comparative analyses	Tyakht et al. (2012)	http://malina.metagenome.ru/
METAGENassist	Various: taxonomic annotation, comparative analyses	Arndt et al. (2012)	http://www.metagenassist.ca/
MetaPhlAn (Galaxy)	Marker gene analysis: similarity	Segata et al. (2012)	http://huttenhower.sph.harvard.edu/galaxy/
NBC	Binning: compositional classification	Rosen et al. (2011)	http://nbc.ece.drexel.edu
Orphelia	Gene prediction	Hoff et al. (2009)	http://orphelia.gobics.de/
Phylopithia webserver	Binning: compositional classification	Patil et al. (2012)	http://phylopythias.cs.uni-duesseldorf.de/
Real time metagenomics	Functional annotation	Edwards et al. (2012)	http://edwards.sdsu.edu/rtmg/
WebCARMA	Binning: sequence similarity	Gerlach et al. (2009)	http://webcarma.cebitec.uni-bielefeld.de/
WebMGA	Various: taxonomic and functional annotation	Wu et al. (2011)	http://weizhong-lab.ucsd.edu/metagenomic-analysis

Knowing which analyses to conduct and which tools to apply remain confusing questions for many scientists. The answer depends largely on several variables, including the hypothesis and goals, the experimental design, and the known properties of the community. For example, a researcher that is interested in identifying well-curated metabolic pathways that are overrepresented in a community may elect to use a database optimized for pathway curation, like KEGG or MetaCyc, to annotate their metagenomes. Conversely, researchers interested in counting the total distinct types of proteins may want to use a database that optimizes for phylogenetic diversity. If the community is known to contain phylogenetically diverged lineages relative to genome sequence databases, then it may be better to use taxonomic annotation techniques that are more tolerant of sequence divergence than fragment recruitment methods. If the main objective is to characterize the genome of a relatively abundant organism in the community, then metagenomic assembly may be warranted. Consideration of the assumptions and limitations of the analytical strategies and tools is critical as the improper approach may fail to detect a meaningful signature or, worse, identify a spurious result.

There are many areas where metagenomic analysis can be improved. First, the precision, thoroughness, and throughput of the analytical strategies reviewed here can be increased. Additional analytical methods (e.g., non-coding RNA detection; Weinberg et al., 2009) are also needed. Second, many of the tools that are currently available would benefit from expansion of the diversity of genome sequence databases, which are frequently queried as referential information during metagenomic analysis. Third, infrastructural developments associated with managing and serving sequence data are needed. Given the plummeting costs of DNA sequencing, it is realistic for researchers to generate massive metagenomes across a large number of samples. The rapid growth in the size of data complicates its storage, organization, and distribution. Fourth, improved statistical methodology is needed, especially for metagenomes generated from complex communities where data for any given taxon or protein may be sparse. Statistical methodology can also improve the identification of biomarkers from comparative studies where a large number of covariates (e.g., environmental or host physiological parameters) are collected for each sample. Finally, additional experimental systems that provide opportunities to manipulate communities, especially microbiota, are needed. Because the results identified through the comparison of metagenomes are typically associative, most studies only produce hypotheses about how communities interact with their environment. Modulating the community composition (e.g., antibiotic administration, gnotobiotic hosts, probiotic supplementation, community transplantation, mono-association of specific taxa) and evaluating the effect on the environment or host provides a direct test of these hypotheses. By coupling metagenomics with this experimental framework across a diverse array of systems, insight into the general rules and properties of community-environment interaction can be gleaned.

## Conflict of Interest Statement

The author declares that the research was conducted in the absence of any commercial or financial relationships that could be construed as a potential conflict of interest.
